# The SLC36 transporter Pathetic is required for neural stem cell proliferation and for brain growth under nutrition restriction

**DOI:** 10.1186/s13064-020-00148-4

**Published:** 2020-08-02

**Authors:** Shiyun Feng, Evanthia Zacharioudaki, Kat Millen, Sarah J. Bray

**Affiliations:** Department of Physiology Development and Neuroscience, University of Cambridge, Downing Street, Cambridge, CB2 3DY UK

**Keywords:** Amino acid transporter, SLC36, Pathetic, Neural stem cells, Neuroblasts, Notch signalling, Nutrition restriction

## Abstract

**Background:**

*Drosophila* neuroblasts (NBs) are neural stem cells whose maintenance relies on Notch activity. NBs proliferate throughout larval stages to generate a large number of adult neurons. Their proliferation is protected under conditions of nutrition restriction but the mechanisms responsible are not fully understood. As amino acid transporters (Solute Carrier transporters, SLCs), such as SLC36, have important roles in coupling nutrition inputs to growth pathways, they may have a role in this process. For example, an SLC36 family transporter Pathetic (Path) that supports body size and neural dendrite growth in *Drosophila*, was identified as a putative Notch target in genome-wide studies. However, its role in sustaining stem cell proliferation and maintenance has not been investigated. This study aimed to investigate the function of Path in the larval NBs and to determine whether it is involved in protecting them from nutrient deprivation.

**Methods:**

The expression and regulation of Path in the *Drosophila* larval brain was analysed using a GFP knock-in allele and reporter genes containing putative Notch regulated enhancers. Path function in NB proliferation and overall brain growth was investigated under different nutrition conditions by depleting it from specific cell types in the CNS, using mitotic recombination to generate mutant clones or by directed RNA-interference.

**Results:**

Path is expressed in both NBs and glial cells in the *Drosophila* CNS. In NBs, *path* is directly targeted by Notch signalling via Su(H) binding at an intronic enhancer, *PathNRE*. This enhancer is responsive to Notch regulation both in cell lines and in vivo. Loss of *path* in neural stem cells delayed proliferation, consistent with it having a role in NB maintenance. Expression from *pathNRE* was compromised in conditions of amino acid deprivation although other Notch regulated enhancers are unaffected. However, NB-expressed Path was not required for brain sparing under amino acid deprivation. Instead, it appears that Path is important in glial cells to help protect brain growth under conditions of nutrient restriction.

**Conclusions:**

We identify a novel Notch target gene *path* that is required in NBs for neural stem cell proliferation, while in glia it protects brain growth under nutrition restriction*.*

## Background

The *Drosophila* Neuroblasts (NBs) are neural stem cells that divide to give progeny, which differentiate into neurons and glia that later constitute the adult brain. NBs arise from neuroectoderm during embryonic development and enter quiescence at the end of the embryonic stage, until they are reactivated upon feeding during larval stages [1, 2]. After reactivation, around 350 NBs reside on the surface of the brain and constitute the stem cell pool, undergoing multiple rounds of asymmetric cell divisions [3]. During division, each NB generates one larger daughter cell that retains stem cell identity and one smaller daughter cell that divides further to generate progeny that differentiate into certain types of neurons and glia [4]. At the time of metamorphosis, the central nervous system (CNS) contains about 30,000 neurons and 10,000 glial cells. The glial cells fulfil supporting and nurturing function to neurons [5]. Importantly they also ensure NBs receive the correct growth signal at the correct times. For example, signals from glia are necessary for NBs to exit quiescence upon feeding [1, 2], and to remain proliferative during nutrition deprivation once the larva passes the critical weight time-point [6].

Notch signalling is one of the key regulators in maintaining NSCs and performs a similar function in both *Drosophila* and vertebrate NSCs. Notch depletion causes loss of NB lineages while Notch over-activation inhibits NBs from differentiating and induces brain tumours [7]. In the canonical Notch signalling model, upon Notch ligand binding to the receptor, the Notch intracellular domain (NICD) is cleaved and released into the nucleus. The nuclear NICD interacts with the DNA-binding protein known as Suppressor of Hairless (Su(H)) in flies, to activate the expression of target genes. The functions of Notch are very context-dependent [8], making it important to identify the Notch regulated genes in different processes including stem cell maintenance.

The brain, like other organs, needs to translate changing nutrition inputs into cell growth decisions. An emerging role of amino acid transporters, especially the SLC family, in coupling the nutrition signalling and growth pathways has been revealed in recent years. SLC38A9 acts as an amino acid sensor in the process of mTORC-activation in mammalian cell lines [9, 10]. Similarly, SLC36A4 helps to promote proliferation in colorectal cancer cells through its interaction with mTORC1 [11]. A requirement for SLC36A4 in mice retinal pigmented epithelial cells also involved mTORC-activation [12]. However, where and how might these transporters function in other cases of nutrient sensing, such as the *Drosophila* NBs, remains unknown. Also, it is unclear whether the growth-promoting role of these amino acid transporters would be adaptive to starvation. For example, the sparing mechanism in nutrient deprived NBs somehow bypasses the Tor pathway [6].

Pathetic (Path) is the *Drosophila* orthologue of SLC36A4, having the characteristics of a broad specificity transporter with multiple transmembrane domains. It interacts with Tor (Target of apamycin) pathway components in regulating eye growth and body growth of *Drosophila* and promotes dendritic growth in C4da neurons [13]. *path* also exhibited the hall marks of a Notch regulated gene in a genome-wide study of genes upregulated during Notch-induced NB hyperplasia [14]. Here, we have followed up on this observation by analysing the role and regulation of Path in NBs under normal and abnormal nutrition conditions. We demonstrate that *path* is indeed a novel direct Notch target in NBs and that it is required for NB proliferation. Further we characterised its role in brain sparing and found that it is required to fully protect the CNS from nutrient restriction. However, our evidence indicates that glial-expressed Path is important for protecting brain growth under nutrient restriction, rather than its activity in the NBs themselves.

## Methods

### Drosophila genetics

*Drosophila* stocks were obtained from the Bloomington Stock Center unless otherwise stated. For RNAi mediated knock down, *UAS-NotchRNAi* (Bloomington 7078) [15] or *UAS-PathRNAi* (Bloomington v100519) were crossed to *insc-Gal4;tubGal80*^*ts*^ (NBs, Bloomington 8751) [16] or *repo-Gal4* (Glial cells) [17]. Notch loss/gain of function in NBs were generated by crossing *insc-Gal4;tubGal80ts* to *Notch-RNAi or NΔECD/CyO,GFP* [14]. *path* mutant clones were generated by crossing *path[KG06640] FRT80B/TM6B* (DGRC111613) [18]) to *hs-FLP,tub-Gal4-UAS-GFP/FM6;;tubGal80FRT80B/TM6B* and inducing FLP mediated recombination by exposing larvae to 37C for 1 h [13, 14]. FRT80B was used to generate control clones. *Path*^*GF*P^ has a GFP insertion immediately upstream of the stop codon in the last coding exon [19].

### Luciferase and NRE reporters

Genomic fragments encompassing Su(H) bound regions in *path* were amplified from Drosophila genomic DNA using primers with restriction site ends (FWD: TAGGGTACCTAAATGCACAGCAACGAAGG; REV: TAGCTCGAGCGATCAAAAGTTCGTTGACC) and subcloned into pGL3min for Luciferase reporters [20] and into pGreenRabbit for in vivo reporter assays [34]. Site-directed mutagenesis was carried out using Pfu Turbo DNA polymerase Kit (Alignment) to mutate high-affinity Su(H) binding sites [21]. For luciferase assays, plasmids were transfected into Drosophila S2 cells and assays carried out as described previously [20].

### Immunofluorescence

Flies were raised at 25^o^C and dissected when they reached the 3rd larval instar, unless otherwise stated. Dissection was performed in pre-chilled PBS and carcasses were then immediately fixed in 4% paraformaldehyde (PFA) for 20 mins, washed 3 times with PBT (PBS with 0.1% Triton X-100), and blocked with PBT with 0.1% BSA for 1 h. Samples were incubated in primary antibody overnight at 4 °C then washed 3 times with PBT and incubated in secondary antibody at room temperature (RT) for 1.5 h. Samples were then washed 3–4 times with PBT and equilibrated in PBS with 70% glycerol overnight before mounting. Brains were mounted in mounting media (Citiflour AF1) for imaging. The following primary antibodies were used: rabbit α-GFP (1:10,000, Sigma), rabbit α-Grh (1:2000, gift of Christos Samakovlis), and rabbit α-Asense (1:2000, courtesy of Y.N.Jan) [22], mouse α-Pros (1:100, Developmental Studies Hybridoma Bank (DSHB)), mouse α-repo (1:500, DSHB), and mouse α-Mira (1:100) [23], guinea-pig α-Dpn (1:5000, gift form C. Delidakis [24];) and α-Path (1:50, [15]), rat α-Elav (1:20 DHSB) Secondary antibodies for mouse, rabbit, guinea pig or rat were conjugated to Alexa 488, 555, 568, 633 or 647 (Molecular Probes) or to FITC, Cy3 or Cy5 (Jackson ImmunoResearch).

### EdU labelling

Brain culturing medium (BCM) was prepared from 10 ml of BCM base solution, consisting of 80% Schneider’s medium, 20% FBS and 10 μl of 10 mg/ml insulin, to which a larval extract (prepared by homogenizing 10 third instar larvae in 200 μl Schneider’s medium and taking the supernatant after centrifugation) was added. Fly brains were dissected in Schneider’s medium and incubated in BCM containing 50 μM of EdU for 4 h at RT. Following EdU incubation, brains were rinsed twice in Schneider’s medium and fixed in 4% PFA for 25 mins at RT. The brains were then rinsed twice in 0.3% PBST, followed by 2 X 20 mins washes in 0.3% PBST. Blocking was carried out by incubating the brains in blocking buffer (0.3% BSA in PBST) for 1 h at RT. After the blocking, the Click-iT reaction was carried out following the instructions in the manual. Brains were rinsed twice in 0.3% PBST and nuclear stain DAPI was included in the penultimate wash. Samples were subsequently mounted in VECTASHIELD anti-fade mounting medium and imaged using point scanning confocal microscopy. For antibody co-staining, this was carried out after the Click-iT reaction and the wash steps, according to the manual.

### Nutrition restriction

In order to test the response of larval brain growth to nutrition restriction, larvae were transferred to a sucrose-only diet regime, 24 h after larval hatching (ALH), with 100–150 larvae per plate. Yeast was added to control plates established in parallel. 96 h–120 h ALH, the brains were dissected from larvae kept under control and sucrose-only conditions.

### Imaging and analysis

Images were taken using Leica SP2 and SP8 confocal microscopes (CAIC, University of Cambridge). Images were processed and labelled in Fiji (ImageJ). Cell counting was performed using the Cell Counter plugin in ImageJ and a cell counting program Counting3D developed by Leila Muresan was used for counting cells in multiple-layer Z-stacks in clonal analysis [14]. Statistics were conducted with Prism6. For pair-wise samples, a t-test was used if the samples fitted a Gaussian distribution, or Kolmogorov-Smirnov test if the samples did not fit Gaussian distribution. For multiple comparison, a one-way ANOVA analysis was used.

## Results

### Path is expressed in neuroblasts and glia

**Fig. 1:**
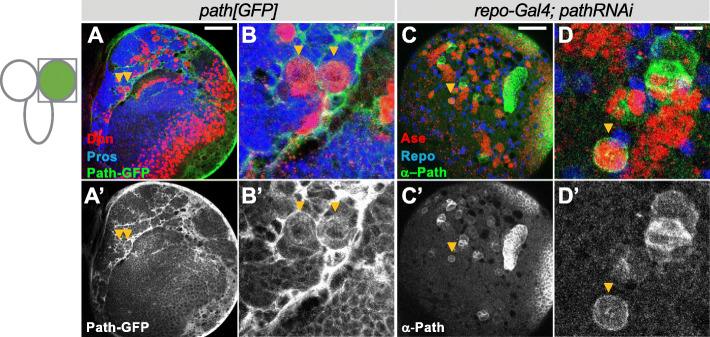
Path is expressed in both glia and neural stem cells. (A-B) Path expression pattern revealed by GFP knock-in allele *path*^*GFP*^ (green in A&B, grey in A’&B′) in the dorsal brain lobe (A), NBs are marked with Dpn (red), and neuronal progeny with Pros (blue). (C-D) NB expression of Path (anti-Path, green in C&D, grey in C′&D’) is visible when glial-expressed Path is depleted by *path-RNAi*. Ase (red) marks NBs and Repo (blue) marks glial cells. Scale bar in A&C, 50 μm; scale bar in B&D, 10 μm

To investigate the function of Path in regulating NB growth and proliferation, its expression in the CNS was firstly analysed using *path*^*GFP*^, a functional allele with GFP inserted at the C-terminus of the Path coding sequence [13]. Path^GFP^ was highly expressed in surface and cortex glia (Fig. 1A & A’), and also, at a much lower level in NBs and their progeny (Fig. 1B & B’ and insets). A similar high level of Path expression in the surface glial cells was also detected with a Path antibody (Additional Fig. S1 [13]), confirming that the protein is present at high levels in these cells. Because this enrichment of Path protein in the surface glia potentially masked expression in the underlying NBs and neurons, we used the glial cell expressing *repo-Gal4*, to down-regulate *path* in glia with RNAi so that we could determine whether Path was expressed in less superficial cells. When the glial expression was suppressed in this way, it was evident that the protein is also present at significant levels in the NB lineages (Fig. 1C & C’). Furthermore, Path expression was enriched in NBs compared to the neuronal progeny (Fig. 1D & D’), in agreement with transcriptomic analysis showing that *path* RNA was enriched in FACS sorted NBs compared to neurons [25]. Together, these data show that Path is expressed in NBs and in glial cells.

### Path is a direct notch target in neural stem cells

**Fig. 2:**
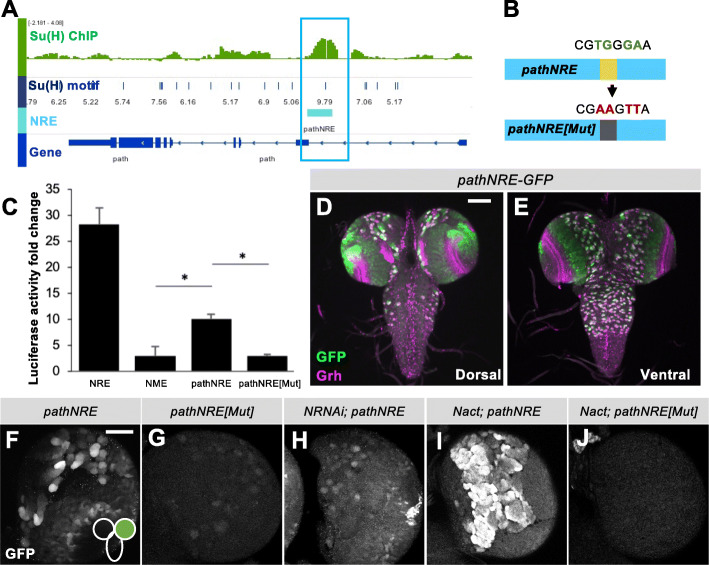
A Notch responsive element (NRE) in *path* directs expression in neural stem cells. (A) A genomic overview of the *path* gene region with the Su(H) binding profile from Nact (*N∆ECD)-*expressing brains. Graph depicts Su(H) bound regions (enrichment relative to input AvgM, scale log2 0–4) in NΔECD brains (green). Blue bars indicate Su(H) binding motifs identified using Patser, height of bar represents Patser score, scale 5–9.79 [26]. Gene models are depicted in blue and cloned *pathNRE* region in cyan. (B) Strategy for mutating high-affinity Su(H) binding motif in *pathNRE*. (C) The response of *pathNRE* to transient activation of Notch in S2 cells. (NRE): positive control, *E*(*spl)m3NRE*, a known Notch target enhancer; (NME): negative control, Notch mutated enhancer (NME). Error bars represent the SD of three biological replicas; *p < 0.05. (D&E) Dorsal (D) and ventral (E) expression from *pathNRE*-GFP reporter detected with anti-GFP in L3 larval brains. Scale bar, 100 μm. (F-J) Reporter expression from the indicated genotypes detected with anti-GFP (green and grey panel). Scale bar, 50 μm

*path* was identified in a genome-wide study as a likely Notch regulated gene in *Drosophila* larval brains, based on its characteristics in conditions where constitutive Notch activity was supplied to NBs causing hyperplasia [14]. *path* expression levels were upregulated in hyperplastic brains produced by constitutive Notch activity in NB lineages [14] and the first intron of *path* exhibited robust Su(H) binding (Fig. 2A), which corresponded to a region with a conserved high-affinity Su(H) binding motif [27]. To find out whether the Su(H) binding of *path* was indicative of direct regulation by Notch, the region encompassing the Su(H) motif, *pathNRE,* was first sub-cloned upstream of a minimal promoter and luciferase reporter (Fig. 2A, cyan). Luciferase activity driven by the *pathNRE* was significantly stimulated in response to activated Notch (Fig. 2C). This response was clearly diminished when the high-affinity Su(H) motif was mutated, *pathNRE[Mut]* (Fig. 2B,C)*.* These results support the hypothesis that the fragment encompasses a Notch-responsive enhancer. Importantly, the fragment has characteristics of a Notch-regulated enhancer in vivo. In transgenic flies, *pathNRE* directed robust GFP expression in NBs as well as part of the optic lobe (Fig. 2D & E), as indicated by the co-staining with the NB marker Grainyhead (Grh). Mutating the Su(H) motif (*pathNRE[Mut])* strongly compromised the enhancer, resulting in a much lower level of GFP expression in NBs and in the optic lobe (Fig. 2G) indicative of direct input from Notch pathway. The fact that expression from *pathNRE[Mut]* in NBs was not completely eliminated, may reflect the loss of the repressive function from Su(H) [28] and suggests that the enhancer is also regulated by other factors.

To assess whether *pathNRE* responds to Notch signalling as predicted, Notch activity in NBs was depleted by expression of *Notch-RNAi*, or enhanced by expression of constitutively active Notch (extracellular domain truncated; N∆ECD). Compared with *pathNRE* control (Fig. 2F), down-regulating *Notch* by RNAi (with *insc-Gal4*, which directs expression in NBs and optic lobe) caused substantial loss of *pathNRE*-GFP expression from most NBs (Fig. 2H) and also reduced levels of Path detectable by antibody (Additional Fig. S2). Conversely, higher levels of *pathNRE*-GFP expression were observed when excessive Notch was generated by expressing N∆ECD in a similar manner (Fig. 2I). In contrast, there was no increase in *pathNRE[Mut]* expression when it was exposed to similar conditions (Fig. 2J), in agreement with it having lost the ability to respond to Notch. Altogether the above results support the hypothesis that *path* has direct input from Notch signalling, via *pathNRE*.

### Path is required for NSC proliferation

**Fig. 3:**
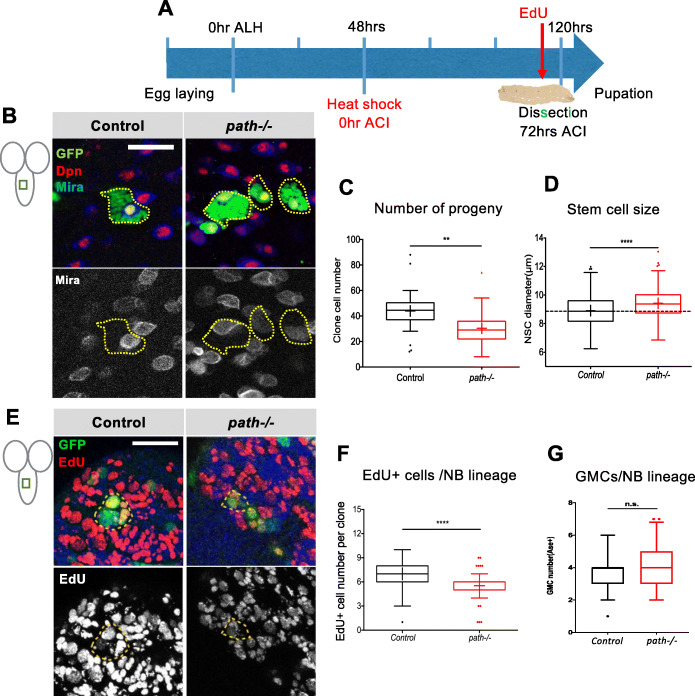
Depletion of Path causes an increase in NSC cell size and a reduction in proliferation. (A) Scheme for mosaic clone induction. Clones were induced (heat-shock treatment) 48 h after larval hatching (ALH) and larvae dissected 72 h after clone induction (ACI). (B) Mosaic clones from control and *path* mutants. NBs are marked with nuclear Dpn (red) and membrane associated Mira (blue & grey). GFP-marked NB lineages are outlined with yellow dotted lines. (C) Number of progeny per labelled lineage, *n* = 26, 42. (D) Quantification of NB sizes, measured using Mira, from controls and *path* mutants; *n* = 253, 120. (E) EdU labelling of control and *path* mutant NB clones 3 days ACI. Cells undergoing DNA synthesis during 4 h of EdU incubation are labelled (red and grey panels); Nuclei are marked by Hoechst (blue) staining. GFP-marked NB lineages are outlined with yellow dotted lines. (F) Quantification of EdU-positive (EdU+) cells in marked NB lineages; *n* = 61, 51. (G) Quantification of GMCs (Asense +ve) in marked NB lineages. **p < 0.01, ***p < 0.001, ****p < 0.0001. Scale bar, 50 μm

Given the role of Notch in maintaining NBs and the evidence that *path* is directly regulated by Notch, it was plausible that Path could play an important role in implementing Notch function in NBs. As Notch is important for survival and proliferation of NBs, the role of *path* in NB proliferation was examined, using the MARCM system to generate random clones of WT and *path* mutant cell lineages marked with GFP (Fig. 3A). Analysing cell number revealed that, when *path* was removed, the NB clones contained less progeny, i.e. fewer cells were present per clone (Fig. 3B & C; mean cell number in control clones = 3.88 ± 3.34, *n* = 26; mean in *path* mutant clones = 30.33 ± 1.89, *n* = 42). At the same time, the mutant stem cells were larger than control ones (Fig. 3B & D, mean size of control NB = 8.91 ± 0.07 μm, *n* = 253; mean size of *path* mutant NB = 9.43 ± 0.11 μm, *n* = 120). The increase in NB size was also observed when *path* was depleted specifically in NBs, by driving *path* RNAi with *insc-Gal4* (Additional Fig. S3A-E, mean size of control NB = 8.71 ± 0.067, *n* = 303; mean size of *path* mutant NBs = 9.58 ± 0.068, *n* = 340). Despite these changes, the *path* mutant NBs retained expression of Deadpan (Dpn) and Miranda (Mira), which are characteristic of these stem cells (Fig. 3B, Additional Fig. S3A-D).

The reduction in cell numbers within NB clonal lineages in the absence of Path suggests either that NBs underwent fewer cell divisions or that more cell death occurred in the progeny. Considering that larger stem cell size can also result from a delay in the cell cycle, changes in proliferation seemed the more likely explanation. Incorporation of EdU (5-ethynyl-2′-deoxyuridine), a thymidine analogue, was therefore measured to estimate the number of cells undergoing DNA replication in WT and *path*-mutated NBs, during a 4 h period (Fig. 3A & E). Under these conditions, *path* mutant clones had fewer cells marked by EdU than control clones (Fig. 3E & F, mean of control EdU+ cells, 6.0 ± 0.22, *n* = 61; mean of EdU+ cells in *path* clones, 4.5 ± 0.25, *n* = 51). This result suggests that the *path* mutant cells divide more slowly. The estimated division rate for control NBs is 85 min and for *path* mutant NBs is 111 min. Furthermore, there was no decrease in the number of GMCs, as might be expected if there was significant cell death (Fig. 3G). In summary, these results suggest a requirement for *path* in maintaining the normal rate of NB proliferation, although an increase in the death of neuronal progeny cannot be ruled out. It remains to be established whether Path plays a similar role in maintaining proliferation in Notch induced tumours where the *path* transcript is up-regulated.

### Path is required for protecting brain growth under nutrition restriction

After larvae reach a critical weight, which occurs approximately 60 h after larval hatching (ALH)), nutrition levels no longer restrain their ability to pupate [29]. As a result, starvation after this stage results in a smaller animal with generally smaller organs, except that the brain achieves a similar size to control counterparts, so-called brain sparing [6]. The Alk/Jeb pathway appears to co-ordinate brain sparing, bypassing Tor and taking the place of Insulin Receptor to activate the PI3K pathway [6]. Sensing of amino acid levels is likely to rely on mechanisms for transporting amino acids into the NB and glial cells and, as Path has characteristics of an amino acid transporter, it could thus play a role in the brain sparing mechanism.

**Fig. 4:**
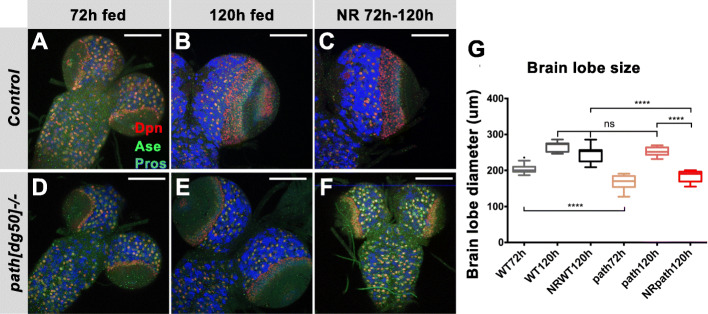
Path is required for brain sparing. (A-F) Control (A-C) and *path[dg50]−/−* (D-F) brains from larvae reared under the indicated conditions: 72 h ALH fed (A&D), 120 h ALH fed (B&E) and NR from 72 h to 120 h ALH (C&F). NBs are marked by Dpn (red), NBs and GMCs are marked by Ase (green) whereas neurons are marked by Pros (blue (G) Quantification of brain lobe diameters in each of the above conditions.). n = 6–10 brains. ****p < 0.0001, ns, not significant (*p* > 0.05). Scale bar, 100 μm

To investigate, we examined whether *path* is required for brain growth under nutrition restriction (NR). Larvae were transferred to a sucrose-only NR diet at early L3 (72 h ALH), after the critical weight time point. Under these NR conditions, the brains of wild-type exhibited a similar growth trajectory to those of fed larvae (Fig. 4C & G). Strikingly, in animals with reduced *path*, namely those homozygous for a hypomorphic allele, *path[dg50]* [13], NR resulted in a greatly reduced brain size (Fig. 4F & G). These data suggest therefore that *path* is involved in the brain-sparing mechanism.

**Fig. 5:**
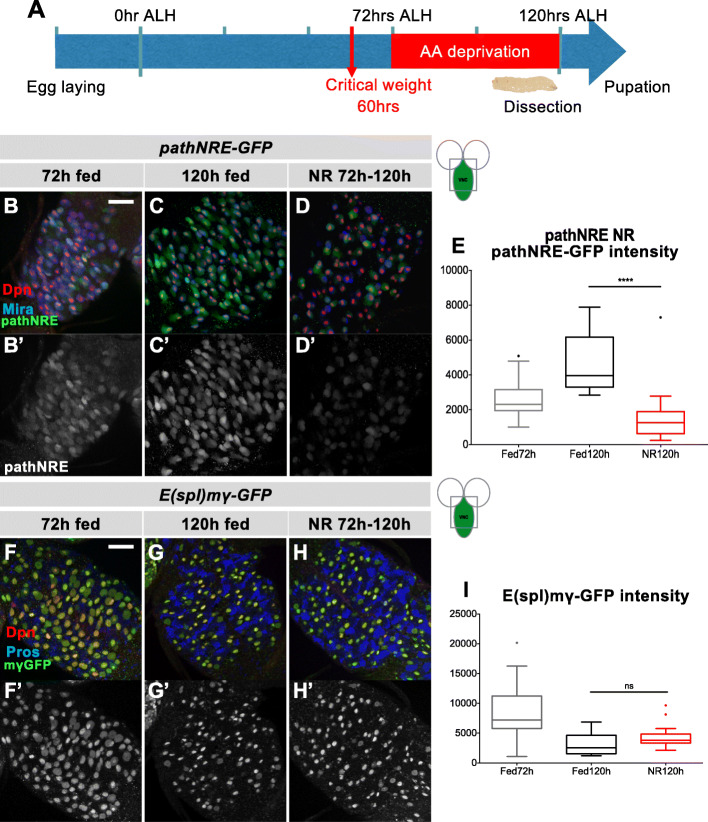
Effects of nutrition restriction on pathNRE and E(spl)mγ-GFP expression. (A) Scheme for experiments with nutrition restriction (NR). Larvae were transferred to amino acid-deprived diet at 72 h (ALH) and dissected at 120 h ALH. (B-E) *pathNRE* expression is reduced under NR conditions. *pathNRE-GFP* (green, grey panel) expression in VNC at 72 h ALH (B), 120 h ALH fed with yeast-rich food (C) and 120 h under NR from 72 h to 120 h ALH (D). NBs are marked with Dpn (red) and Mira (blue). (E) Quantification of *pathNRE-GFP* intensity in above conditions (*n* = 18,16,22). (F-I) Neural stem cells retain Notch activity in response to NR. (F-H) Notch regulated *E(spl)mγ-GFP* (green, grey panel) expression in VNC at 72 h ALH (F), 120 h ALH fed (G) and 120 h with NR from 72 h to 120 h ALH (H). NBs are marked with Dpn (red), neuronal progeny are marked with Pros (blue). (I) Quantification of E*E*(spl)mγ-GFP intensity in above conditions (*n* = 15,14,20). ****p < 0.0001; ns, not significant (p > 0.05). Scale bar, 50 μm

To investigate whether the regulation of *path* during brain sparing occurs via *pathNRE,* brains were dissected from nutrient restricted conditions and *pathNRE* expression compared with that of fed larval brains of an equivalent age (Fig. 5A). In normal conditions, *pathNRE* expression became detectable following NB reactivation (Additional Fig. S4) so that it was expressed in all NBs by late L2 (Fig. 5B) and then achieved high levels by 120 h ALH (Fig. 5C & E). Notably, *pathNRE* expression was significantly reduced upon starvation (Fig. 5C, D & E), although the brain size was similar to that of fed larvae, indicating that brain sparing was occurring.

To clarify whether the decrease in *pathNRE* expression was because of alterations in Notch activity, expression of *E(spl)mγ-GFP*, a widely used Notch activity indicator [30, 31], was also examined under NR conditions. Unlike *pathNRE*, *E(spl)mγ-GFP* expression levels under NR were similar to fed larvae at 120 h ALH (Fig. 5F-I). This suggests that Notch activity per se is not changed by NR in larval NBs so that the reduction of *pathNRE* expression in NR conditions is specific. This reduction may indicate that NB expression of Path is not required during starvation. Alternatively, a separate enhancer could operate under these conditions to confer *path* NB expression. Certainly there was not a widespread decrease in path expression after NR, as the expression of *path[GFP]* remained high in glia (Additional Fig. S5A-F).

### Glia-expressed path is required for protecting brain growth under nutrition restriction

**Fig. 6:**
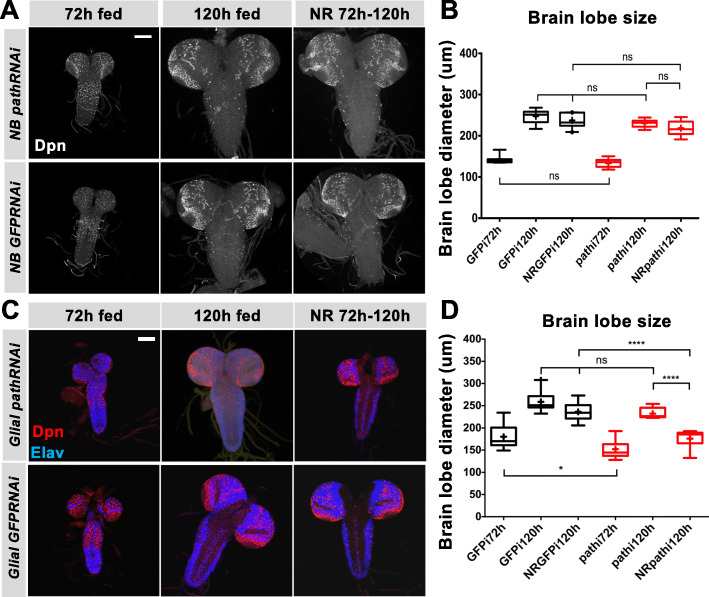
Glial Path expression is required for brain sparing. (A) Brains with *insc-Gal4* driving *path-RNAi* or *GFP-RNAi* as indicated in NBs from 72 h ALH fed, 120 h ALH fed and NR from 72 h to 120 h ALH. Dpn (white) marks NBs. (B) Quantification of brain lobe diameter under indicated conditions. (C) Brains with *repo-Gal4* driving *path-RNAi* or *GFP-RNAi* in glial cells from 72 h ALH fed, 120 h ALH fed and NR from 72 h to 120 h ALH. Dpn (red) marks NBs and Elav (blue) marks neurons (D) Quantification of brain lobe diameter under indicated conditions. n = 6–10 brains. ****p < 0.0001, ns, *p* > 0.05. Scale bar, 100 μm

To elucidate in which cell type Path is required for brain sparing, *path* was first knocked down specifically in NBs that were subject to NR from 72 h ALH onwards. The brain size following NR was comparable with that from fed larvae (Fig. 6A & B), suggesting that *path* expression in NBs was not required for brain sparing during NR. Second, *path* was specifically silenced in glial cells, using *repo-Gal4*. Path depletion alone did not affect brain size under normal conditions, as the fed *pathRNAi* brains had a comparable size to those from fed control larvae (Fig. 6C & D). However, when *pathRNAi* larvae were subject to NR from 72 h ALH, the brains did not grow to the same extent as those from fed larvae, and were more comparable in size to those from much younger larvae (Fig. 6C & D). This suggests that the brain growth is no longer spared when Path is absent from glial cells. Taken together, these results suggest that NB-expressed Path does not participate in protecting brain growth during NR, while the expression of *path* in glial cells is important for brain sparing.

## Discussion

**Fig. 7:**
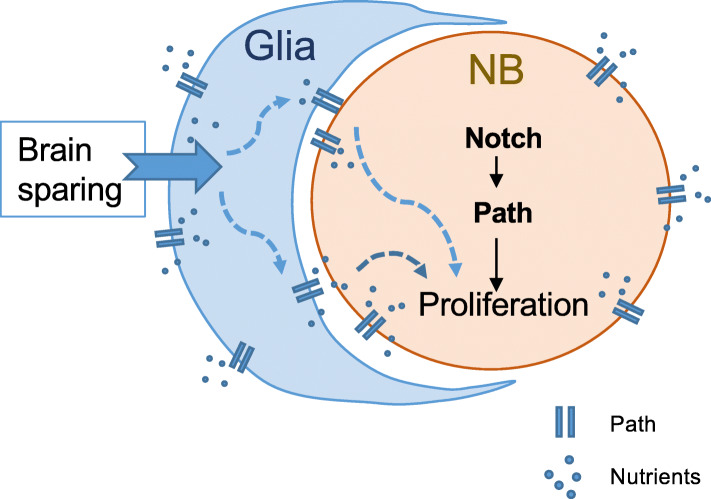
Model of *path* function

Previously, Path was found to be required for overall body growth and extreme dendrite growth, potentially through interacting with the PI3K/Tor pathway and protein synthesis pathways [13]. Here we identified an autonomous role of *path* in maintaining NB proliferation, which is in line with previous findings that *path* promotes growth. Path expression in NBs is partially dependant on Notch, as it contains an intronic enhancer, which is directly regulated by the pathway. Thus its regulation and involvement in NB proliferation argues that Path contributes to the normal function of Notch in NBs (Fig. 7), although it remains to be established whether it has a similar essential role in Notch induced tumours. Furthermore, the striking reduction in *pathNRE* driven expression under NR suggests that the NBs are sensitive to changes in the internal milieu of the animal. This argues that, while glial cells may shield NBs from many environmental effects, the NBs are nevertheless able to detect altered nutrient levels and may harbour addition pathways that contribute to brain-sparing.

Although we find that Path is required for brain sparing during NR, this appears to rely on its expression in Glial cells rather than NBs, despite the fact that the intronic *pathNRE* enhancer is sensitive to nutrient deprivation. Alk/Jeb is the major pathway that has been linked to brain growth under NR conditions, and glial knockdown of Jeb (*repo-gal4 > jebRNAi*) resulted in smaller NB-clone size as well as lineage number [6]. It is possible that Path at the membrane of surface glial cells could detect the environmental amino acid levels and in turn regulate Alk/Jeb expression. Path is a potential amino acid transporter with multiple transmembrane domains, which exhibited high affinity for alanine and glycine with low transporting capacity when expressed in *Xenopus* oocytes [18]. The closest mammalian relatives, proton-assisted transporter 4 (PAT4 or hPAT4), have a high affinity for proline and tryptophan [32]. Although it remains to be fully determined which are the main substrates for Path in vivo, a recent study suggests that Path is required for scavenging proline to promote tumour growth in conditions of obesity enhanced tumorigenesis in *Drosophila* [33]. In these conditions, Path regulation was also involved with nutrient sensing mechanisms albeit the levels of sugars were elevated rather than restricted. Further studies will be needed to determine whether proline is the primary substrate for Path in all conditions and the extent that *path* expression is differentially regulated by changes in nutrient levels according to the tissue and its proliferative state.

The Tor pathway is involved in NB reactivation, growth and proliferation [2]. However, in L3, NB growth is regulated by the Alk/Jeb pathway which activates downstream PI3K/Akt signalling, but appears independent of Tor [6]. One model to explain the current results is that Path functions as a mediator between Alk and the Tor pathway. Alk is required for and acts through the PI3K/Akt pathway. In L3 larvae brains, most Tor pathway components are dispensable for brain growth and brain size is not changed significantly when levels of Insulin-like peptides (Ilp) are manipulated [6]. In contrast, the Tor pathway is activated in younger brains suggesting there may be a mechanism to bypass or switch-off the Tor pathway at later stages. One hypothesis, considering *pathNRE* is upregulated after NB reactivation (Fig. 4.9B&C), is that *path* helps to keep activity of the Tor pathway at restricted levels at later stages. In this case, the effects of *path* in NBs would be through nutrient sensing and protein synthesis, similar to its function in C4da neurons [13].

The regulation and expression of Path in the NBs suggests that, in this context, Path promotes cell growth and lineage size. Further studies will be needed to characterise what the consequences from Path up-regulation are in different circumstances. For example, whether Path has the same role in Notch induced NB tumours, and whether this would differ depending on nutrient status, remains to be established. Likewise, our study has investigated its role in so-called Type I NBs. We did not obtain enough mutant clones to distinguish whether it makes the same contribution in Type II NBs, which produce a highly proliferative transit amplifying intermediary, or whether its activity there differs. Finally, it is striking that pathNRE-GFP expression is decreased in neuroblasts under nutrient restriction, when neuroblast proliferation becomes spared. Whether this reflects a physiological response, whereby NB Path levels are reduced while glial cell Path persists under NR, or whether it reflects a regulatory feature, whereby a different enhancer becomes activated in these conditions, remains to be established. Nevertheless, these data highlight the importance of distinguishing the different regulatory inputs to Path, and the extent that they are dependent on different nutrient and/or amino acid availabilities.

## Conclusion

Brain expression of Path, which encodes a broad specificity transporter, occurs in both NBs and glial cells where its functions appear to differ. NB expression of *path,* is partially dependant on Notch activity and is required for NB lineage proliferation but not for brain sparing under nutrient restrictions. In contrast Glial expression of Path appears to be independent of Notch and is essential for protecting the brain growth under nutrition restriction. In conclusion, we demonstrate different roles of Path in distinct parts of the brain that would together enable the larval brain to proliferate and grow in both normal and NR conditions.

## Supplementary information

**Additional file 1 Fig. S1.** Path is expressed in both glia and neural stem cells. (A-D) Anti-Path staining (green in A&B, grey in A’&B′) is enriched at the surface of the brain, as shown on the same surface with glial cells marked by Repo (magenta). (E-F) Anti-Path (green in C&D, grey in C′&D’) stains NBs when glial-expressed Path is depleted by *pathRNAi*. TypeI NBs are marked with Ase (red), glia cells were marked with Repo (blue) Scale bar, 50 μm.

**Additional file 2 Fig. S2**. Path depletion leads to a reduction in NB size. (A-D) Control (A&B) and *path* knockdown (C&D) brains (*path-RNAi* driven by NB-specific *insc-Gal4*), larvae were incubated at 30 °C for 5 days before dissection. Central brain dorsal (A&C) and VNC (B&D) brain NBs are marked with Dpn (red) and Mira (green). CB, central brain; OL, optic lobe. (E) Quantification of NB size with GFP-RNAi and path-RNAi. *n* = 303, 340. ****p < 0.0001. Scale bar, 50 μm.

**Additional file 3 Fig. S3.** Path[GFP] persists under NR condition. Path[GFP] expression in the indicated conditions: 72 h ALH fed (A&B), 120 h ALH fed (C&D) and NR from 72 h to 120 h ALH (E&F). (A,C,E) dorsal surface, brain lobes (A’,C′,E’) middle-plane, brain lobes, (A”,C″,E”) ventral surface, brain lobes. (B,D,F) ventral surface, VNC. NBs are marked with Dpn (red), neurons are marked with Pros (blue). Scale bar, 100 μm.

**Additional file 4 Fig. S4.** Path[NRE} expression is detected in NBs that have exited quiescence. Expression of Path[NRE]-GFP is only detected in a subset of Dpn and Miranda expressing NBS at 24 h ALH. Small quiescent and recently re-activated NBs have not yet initiated expression (e.g. white arrowheads).

**Additional file 5 Fig. S5:** Path[GFP] persists under NR condition. Path[GFP] expression in the indicated conditions: 72 h ALH fed (A&B), 120 h ALH fed (C&D) and NR from 72 h to 120 h ALH (E&F). (A,C,E) dorsal surface, brain lobes (A’,C′,E’) middle-plane, brain lobes, (A”,C″,E”) ventral surface, brain lobes. (B,D,F) ventral surface, VNC. NBs are marked with Dpn (red), neurons are marked with Pros (blue). Scale bar, 100 μm.

## Data Availability

All data generated or analysed during this study are included in this published article and its supplementary information files. Raw images used for quantifications and materials generated are available from the corresponding author on reasonable request.

## References

[1] Chell JM, Brand AH (2010). Nutrition-responsive glia control exit of neural stem cells from quiescence. Cell.

[2] Sousa-Nunes R, Yee LL, Gould AP. Fat cells reactivate quiescent neuroblasts via TOR and glial insulin relays in Drosophila. Nature. 2011;471(7339):508–12. 10.1038/nature09867 PMC3146047 21346761

[3] Homem CCF, Knoblich JA. Drosophila neuroblasts: a model for stem cell biology. Development. 2012;139(23):4297–310. 10.1242/dev.080515 23132240

[4] Boone JQ, Doe CQ. Identification of Drosophila type II neuroblast lineages containing transit amplifying ganglion mother cells. Dev Neurobiol. 2008;68(9):1185–95. 10.1002/dneu.20648 PMC2804867 18548484

[5] Schirmeier S, Matzat T, Klämbt C. Axon ensheathment and metabolic supply by glial cells in Drosophila. Brain Res. 2016;1641(Pt A):122–29. 10.1016/j.brainres.2015.09.003 26367447

[6] Cheng LY, Bailey AP, Leevers SJ, Ragan TJ, Driscoll PC, Gould AP (2011). Anaplastic lymphoma kinase spares organ growth during nutrient restriction in Drosophila. Cell.

[7] Wang H, Somers GW, Bashirullah A, Heberlein U, Yu FW, Chia W (2006). Aurora-a acts as a tumor suppressor and regulates self-renewal of Drosophila neuroblasts. Genes Dev.

[8] Bray SJ (2016). Notch signalling in context. Nat Rev Mol Cell Biol.

[9] Wang S, Tsun ZY, Wolfson RL, Shen K, Wyant GA, Plovanich ME et al. Lysosomal amino acid transporter SLC38A9 signals arginine sufficiency to mTORC1. Science. 2015;347(6218):188–94. 10.1126/science.1257132 PMC4295826 25567906

[10] Rebsamen M, Pochini L, Stasyk T, de Araújo ME, Galluccio M, Kandasamy RK, et al. SLC38A9 is a component of the lysosomal amino acid sensing machinery that controls mTORC1. Nature. 2015;519(7544):477–81. 10.1038/nature14107 PMC4376665 25561175

[11] Fan S-J, Snell S, Turley H, Li T-L, McCormick R, Perera SMW, et al. PAT4 levels control amino-acid sensitivity of rapamycin-resistant mTORC1 from the Golgi and affect clinical outcome in colorectal cancer. Oncogene. 2016;35(23):3004–15. 10.1038/onc.2015.363 PMC4705441 26434594

[12] Shang P, Valapala M, Grebe R, Hose S, Ghosh S, Bhutto IA, et al. The amino acid transporter SLC36A4 regulates the amino acid pool in retinal pigmented epithelial cells and mediates the mechanistic target of rapamycin, complex 1 signaling. Aging Cell. 2017;16(2):349–59. 10.1111/acel.12561 PMC5334531 28083894

[13] Lin W-Y, Williams C, Yan C, Koledachkina T, Luedke K, Dalton J, et al. The SLC36 transporter pathetic is required for extreme dendrite growth in Drosophila sensory neurons. Genes Dev. 2015;29(11):1120–35. 10.1101/gad.259119.115 PMC4470281 26063572

[14] Zacharioudaki E, Housden BE, Garinis G, Stojnic R, Delidakis C, Bray SJ. Genes implicated in stem cell identity and temporal programme are directly targeted by notch in neuroblast tumours. Development. 2016;143(2):219–31. 10.1242/dev.126326 PMC4725341 26657768

[15] Presente A, Shaw S, Nye JS, Andres AJ. Transgene-mediated RNA interference defines a novel role for notch in chemosensory startle behavior. Genesis. 2002;34(1–2):165–9. 10.1002/gene.10149 12324975

[16] Luo L, Joyce Liao Y, Jan LY, Jan YN (1994). Distinct morphogenetic functions of similar small GTPases: Drosophila Drac1 is involved in axonal outgrowth and myoblast fusion. Genes Dev.

[17] Sepp KJ, Schulte J, Auld VJ. Peripheral glia direct axon guidance across the CNS/PNS transition zone. Dev Biol. 2001;238(1):47–63. 10.1006/dbio.2001.0411 11783993

[18] Goberdhan DCI, Meredith D, Boyd CAR, Wilson C (2005). PAT-related amino acid transporters regulate growth via a novel mechanism that does not require bulk transport of amino acids. Development.

[19] Lin W, Williams C, Yan C, Parrish JZ. Functions of the SLC36 transporter Pathetic in growth control. Fly(Austin). 2019;1:1689–99. 10.1080/19336934.2015.1129089 PMC4862433 26735916

[20] Krejčí A, Bray S (2007). Notch activation stimulates transient and selective binding of Su(H)/CSL to target enhancers. Genes Dev.

[21] Terriente-Felix A, et al. Notch cooperates with lozenge/Runx to lock haemocytes into a differentiation programme. Development. 2013;140(4):926–37. 10.1242/dev.086785 PMC3557782 23325760

[22] Brand M, Jarman AP, Jan LY, Jan YN. Asense is a Drosophila neural precursor gene and is capable of initiating sense organ formation. Development. 1993;119(1):1–17.10.1242/dev.119.Supplement.18565817

[23] Ohshiro T, Yagami T, Zhang C, Matsuzaki F. Role of cortical tumour-suppressor proteins in asymmetric division of Drosophila neuroblast. Nature. 2000;408(6812):593–6. 10.1038/35046087 11117747

[24] Magadi SS, Voutyraki C, Anagnostopoulos G, Zachariuodaki E, Poutakidou IK, Efraimoglou et al. Dissecting Hes-centered transcriptional networks in neural stem cell maintenance and tumorigenesis in Drosophila. bioRxiv. 2020. 10.1101/2020.03.25.007187. 10.1242/dev.191544 33229432

[25] Berger C, Harzer H, Burkard TR, Steinmann J, van der Hoorst S, et al. FACS purification and transcriptome analysis of Drosophila neural stem cells reveals a role for Klumpfuss in self-renewal. Cell Rep. 2012;2(2):407–18. 10.1016/j.celrep.2012.07.008 PMC3828055 22884370

[26] Hertz GZ, Stormo GD. Identifying DNA and protein patterns with statistically significant alignments of multiple sequences. Bioinformatics. 1999;15:563–77. 10.1093/bioinformatics/15.7.563 10487864

[27] Rebeiz M, Reeves NL, Posakony JW. SCORE: a computational approach to the identification of cis-regulatory modules and target genes in whole-genome sequence data. Proc Natl Acad Sci U S A. 2002;99(15):9888–93. 10.1073/pnas.152320899 PMC125053 12107285

[28] Bray SJ, Furriols M. Notch pathway: Making sense of Suppressor of Hairless. Current Biology. 2001;11(6):R217–21. 10.1016/s0960-9822(01)00109-9 11301266

[29] Mirth CK, Shingleton AW. Integrating body and organ size in Drosophila: Recent advances and outstanding problems. Frontiers in Endocrinology. 2012;3:49. 10.3389/fendo.2012.00049 PMC3356080 22654869

[30] Almeida MS, Bray SJ. Regulation of post-embryonic neuroblasts by Drosophila Grainyhead. Mech Dev. 2005;122(12):1282–93. 10.1016/j.mod.2005.08.004 16275038

[31] Zacharioudaki E, Magadi SS, Delidakis C (2012). bHLH-O proteins are crucial for Drosophila neuroblast self-renewal and mediate notch-induced overproliferation. Development.

[32] Muralidharan Pillai S, Meredith D. SLC36A4 (hPAT4) is a high affinity amino acid transporter when expressed in Xenopus laevis oocytes. J Biol Chem. 2011;286(4):2455–60. 10.1074/jbc.M110.172403 PMC3024739 21097500

[33] Newton H, Wang Y-F, Camplese L, Brown AEX, Hirabayashi S. Systemic muscle wasting and coordinated tumour response drive tumourigenesis. bioRxiv. 2019. 10.1101/785022. 10.1038/s41467-020-18502-9 PMC7495438 32938923

[34] Housden BE, Millen K, Bray SJ. Drosophila Reporter Vectors Compatible with ΦC31 Integrase Transgenesis Techniques and Their Use to Generate New Notch Reporter Fly Lines. G3 (Bethesda). 2012;2(1):79–82. 10.1534/g3.111.001321 PMC3276196 22384384

