# Supplementary material for: The SLC36 transporter Pathetic is required for neural stem cell proliferation and for brain growth under nutrition restriction
Source: Neural Dev. 2020 Aug 2;15:10. doi: 10.1186/s13064-020-00148-4 (PMC7398078; doi:10.1186/s13064-020-00148-4)
Supplement: Supplementary file 1 — Additional file 1 Fig. S1. Path is expressed in both glia and neural stem cells. (A-D) Anti-Path staining (green in A&B, grey in A’&B′) is enriched at the surface of the brain, as shown on the same surface with glial cells marked by Repo (magenta). (E-F) Anti-Path (green in C&D, grey in C′&D’) stains NBs when glial-expressed Path is depleted by pathRNAi. TypeI NBs are marked with Ase (red), glia cells were marked with Repo (blue) Scale bar, 50 μm. [file 13064_2020_148_MOESM1_ESM.pdf]

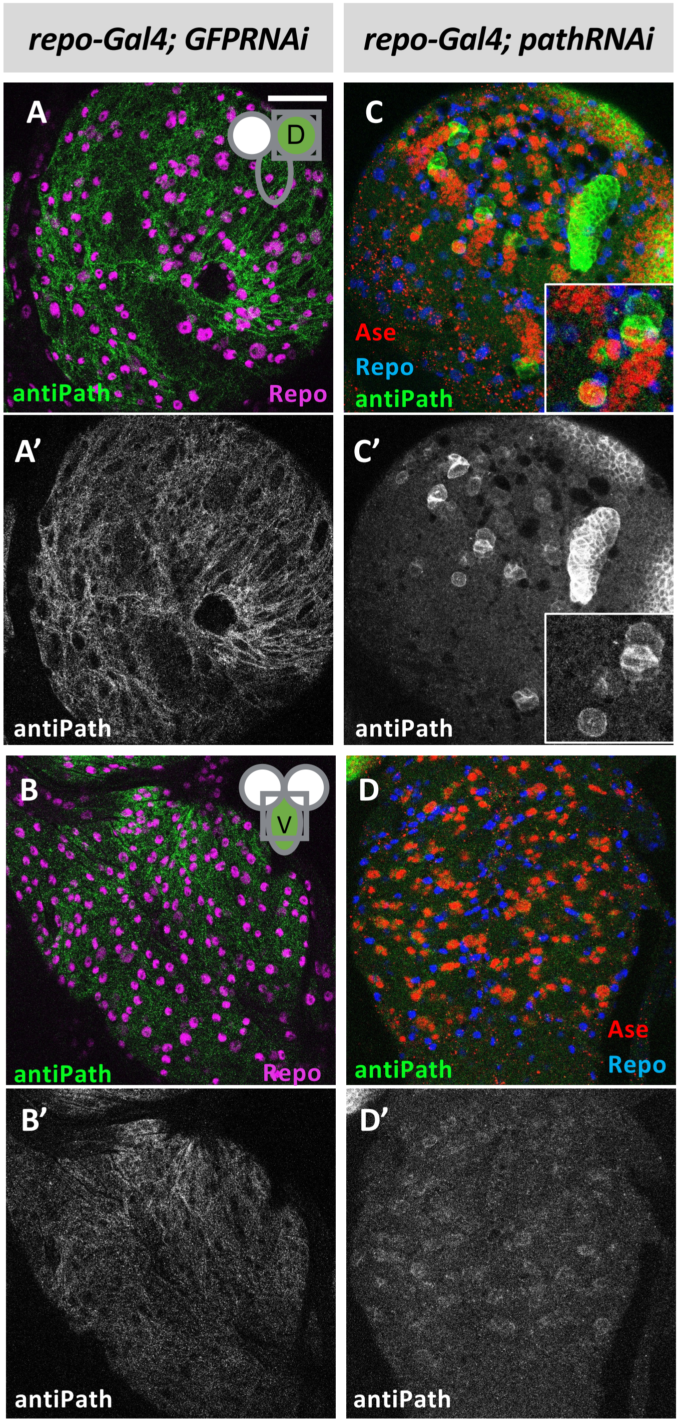

Additional Figure S1. Path is expressed in both glia and neural stem cells. (A-D) Anti-Path staining (green in A&B, grey in A'&B') is enriched at the surface of the brain, as shown on the same surface with glial cells marked by Repo (magenta). (E-F) Anti-Path (green in C&D, grey in C'&D') stains NBs when glial-expressed Path is depleted by *pathRNAi*. TypeI NBs are marked with Ase (red), glia cells were marked with Repo (blue) Scale bar, 50 µm.
