# Supplementary material for: The SLC36 transporter Pathetic is required for neural stem cell proliferation and for brain growth under nutrition restriction
Source: Neural Dev. 2020 Aug 2;15:10. doi: 10.1186/s13064-020-00148-4 (PMC7398078; doi:10.1186/s13064-020-00148-4)
Supplement: Supplementary file 2 — Additional file 2 Fig. S2. Path depletion leads to a reduction in NB size. (A-D) Control (A&B) and path knockdown (C&D) brains (path-RNAi driven by NB-specific insc-Gal4), larvae were incubated at 30 °C for 5 days before dissection. Central brain dorsal (A&C) and VNC (B&D) brain NBs are marked with Dpn (red) and Mira (green). CB, central brain; OL, optic lobe. (E) Quantification of NB size with GFP-RNAi and path-RNAi. n = 303, 340. ****p < 0.0001. Scale bar, 50 μm. [file 13064_2020_148_MOESM2_ESM.pdf]

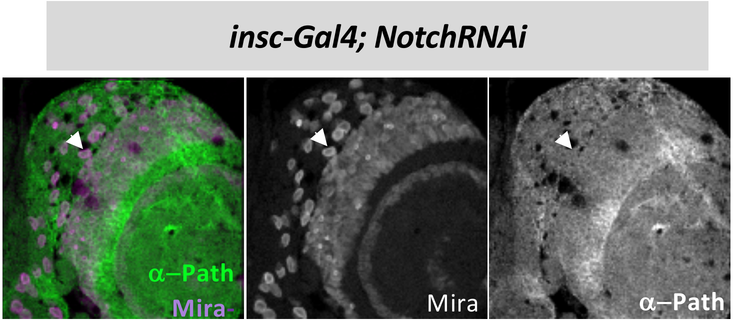

Additional Figure S2. Path in NBs is reduced following Notch RNAi. Exposure to Notch RNAi leads to loss of anti-Path (green) staining from some NBs (e.g. arrowhead), marked by Miranda (purple).
