# Supplementary material for: The SLC36 transporter Pathetic is required for neural stem cell proliferation and for brain growth under nutrition restriction
Source: Neural Dev. 2020 Aug 2;15:10. doi: 10.1186/s13064-020-00148-4 (PMC7398078; doi:10.1186/s13064-020-00148-4)
Supplement: Supplementary file 3 — Additional file 3 Fig. S3. Path[GFP] persists under NR condition. Path[GFP] expression in the indicated conditions: 72 h ALH fed (A&B), 120 h ALH fed (C&D) and NR from 72 h to 120 h ALH (E&F). (A,C,E) dorsal surface, brain lobes (A’,C′,E’) middle-plane, brain lobes, (A”,C″,E”) ventral surface, brain lobes. (B,D,F) ventral surface, VNC. NBs are marked with Dpn (red), neurons are marked with Pros (blue). Scale bar, 100 μm. [file 13064_2020_148_MOESM3_ESM.pdf]

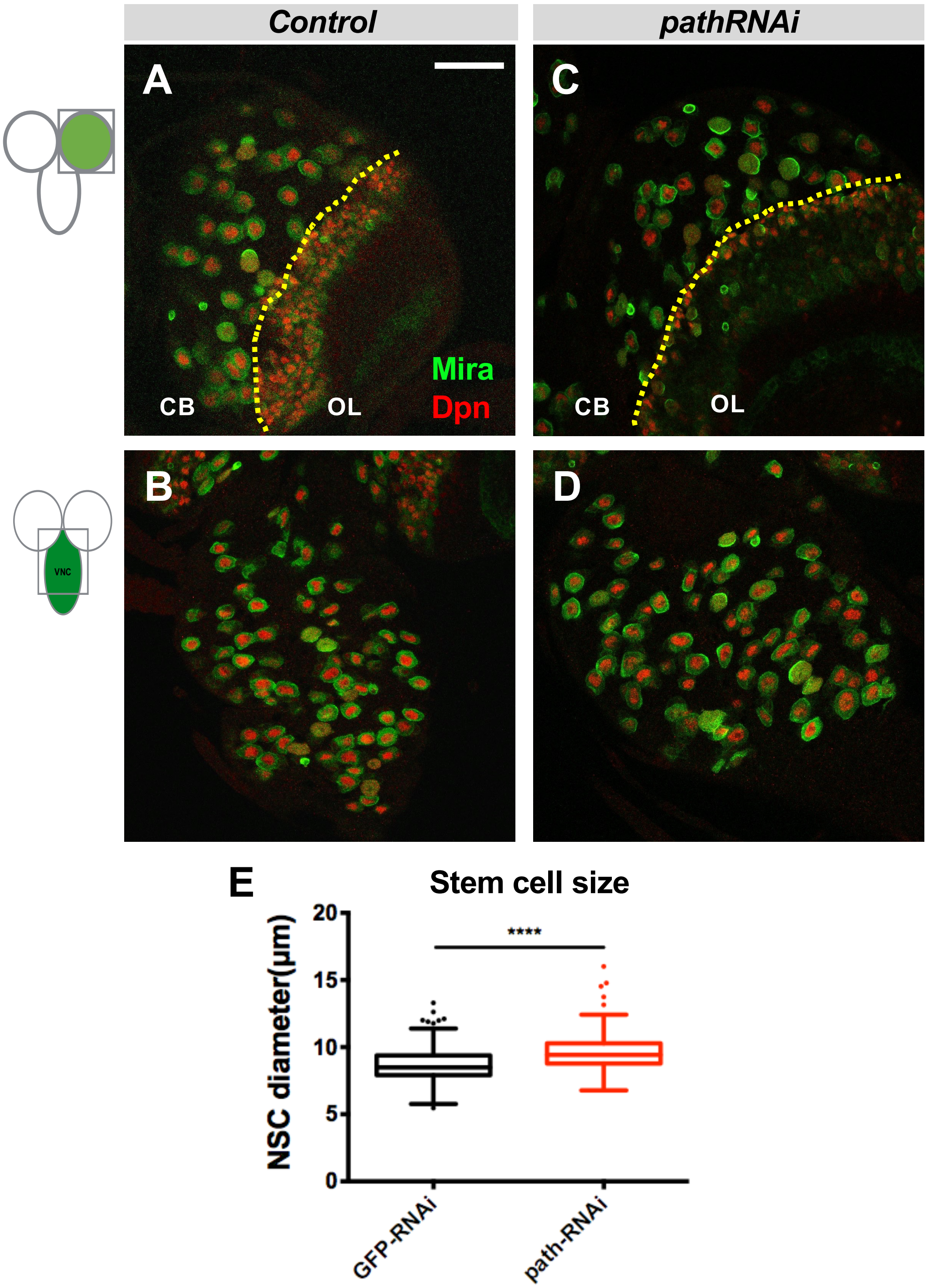

Additional Figure S3: Path depletion leads to a reduction in NB size. (A-D) Control (A&B) and *path* knockdown (C&D) brains (*path-RNAi* driven by NB-specific *insc-Gal4*), larvae were incubated at 30°C for 5 days before dissection. Central brain dorsal (A&C) and VNC (B&D) brain NBs are marked with Dpn (red) and Mira (green). CB, central brain; OL, optic lobe. (E) Quantification of NB size with GFP-RNAi and path-RNAi. n=303, 340. ****$p<0.0001$. Scale bar, 50 μm.
