# Supplementary material for: The SLC36 transporter Pathetic is required for neural stem cell proliferation and for brain growth under nutrition restriction
Source: Neural Dev. 2020 Aug 2;15:10. doi: 10.1186/s13064-020-00148-4 (PMC7398078; doi:10.1186/s13064-020-00148-4)
Supplement: Supplementary file 4 — Additional file 4 Fig. S4. Path[NRE} expression is detected in NBs that have exited quiescence. Expression of Path[NRE]-GFP is only detected in a subset of Dpn and Miranda expressing NBS at 24 h ALH. Small quiescent and recently re-activated NBs have not yet initiated expression (e.g. white arrowheads). [file 13064_2020_148_MOESM4_ESM.pdf]

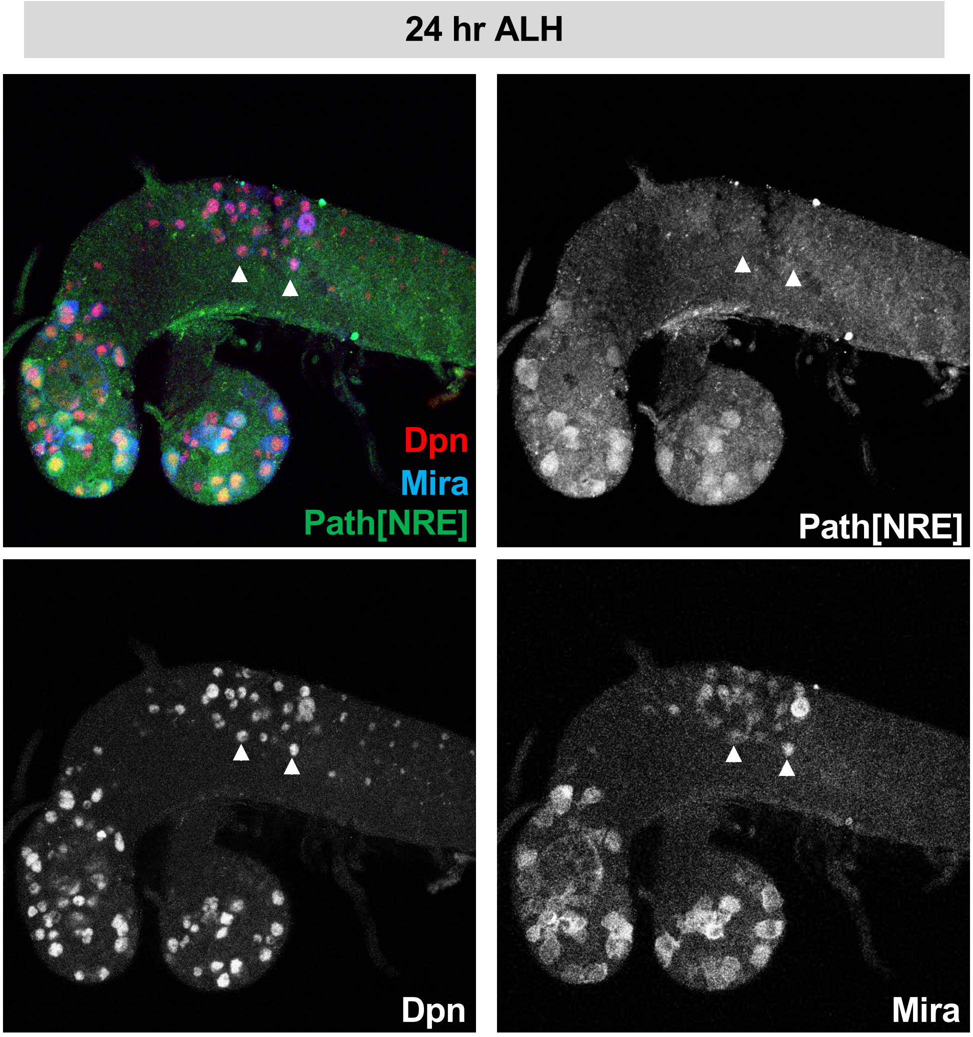

Additional Figure S4: Path[NRE} expression is detected in NBs that have exited quiescence. Expression of Path[NRE]-GFP is only detected in a subset of Dpn and Miranda expressing NBS at 24hr ALH. Small quiescent and recently re-activated NBs have not yet initiated expression (e.g. white arrowheads).
