# Supplementary material for: The SLC36 transporter Pathetic is required for neural stem cell proliferation and for brain growth under nutrition restriction
Source: Neural Dev. 2020 Aug 2;15:10. doi: 10.1186/s13064-020-00148-4 (PMC7398078; doi:10.1186/s13064-020-00148-4)
Supplement: Supplementary file 5 — Additional file 5 Fig. S5: Path[GFP] persists under NR condition. Path[GFP] expression in the indicated conditions: 72 h ALH fed (A&B), 120 h ALH fed (C&D) and NR from 72 h to 120 h ALH (E&F). (A,C,E) dorsal surface, brain lobes (A’,C′,E’) middle-plane, brain lobes, (A”,C″,E”) ventral surface, brain lobes. (B,D,F) ventral surface, VNC. NBs are marked with Dpn (red), neurons are marked with Pros (blue). Scale bar, 100 μm. [file 13064_2020_148_MOESM5_ESM.pdf]

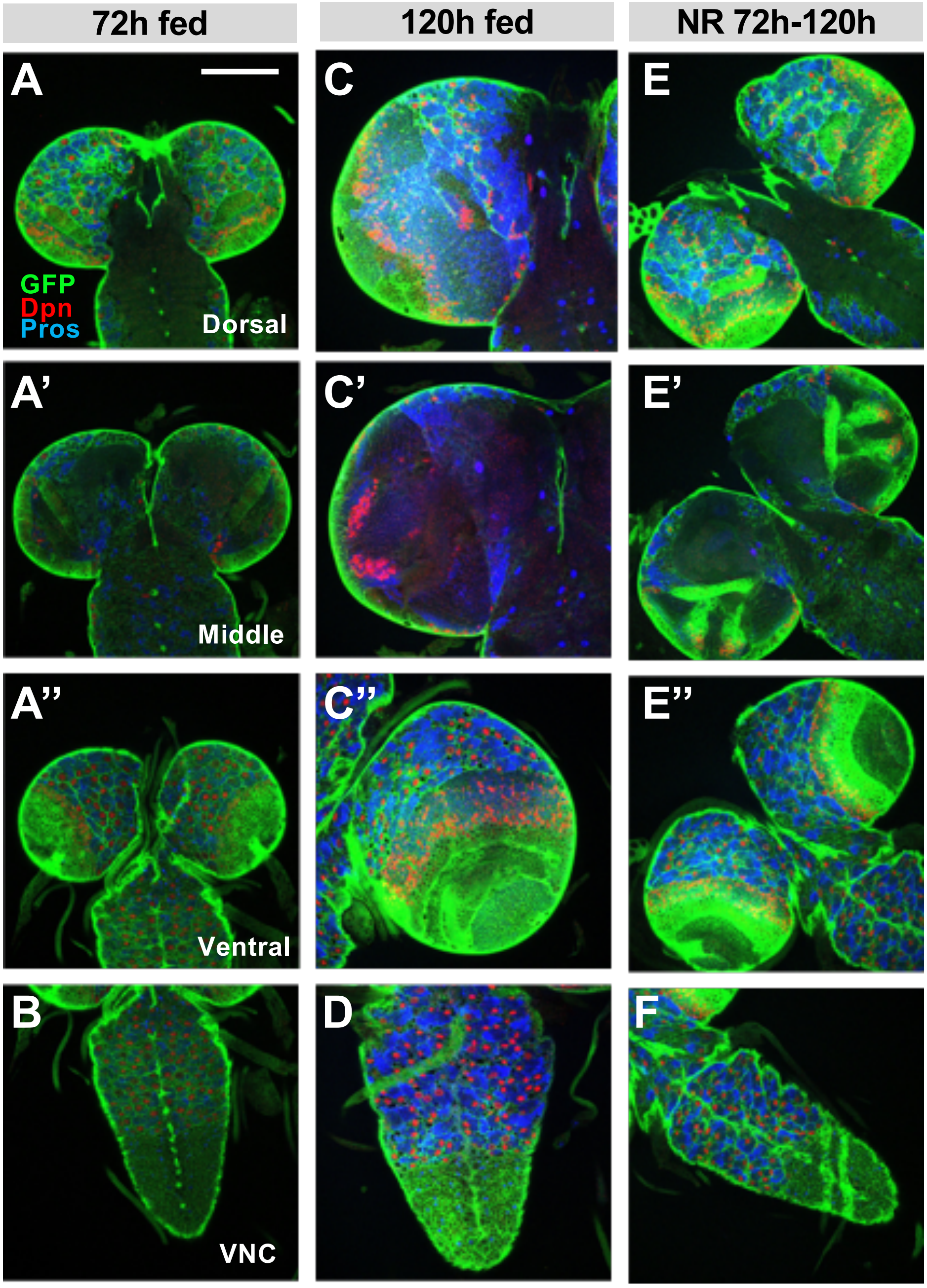

Additional Figure S5: Path[GFP] persists under NR condition. Path[GFP] expression in the indicated conditions: 72hrs ALH fed (A&B), 120hrs ALH fed (C&D) and NR from 72h to 120h ALH (E&F). (A,C,E) dorsal surface, brain lobes (A’,C’,E’) middle-plane, brain lobes, (A’’,C’’,E’’) ventral surface, brain lobes. (B,D,F) ventral surface, VNC. NBs are marked with Dpn (red), neurons are marked with Pros (blue). Scale bar, 100 µm.
